# Mechanical Properties and Chloride Penetration Resistance of Concrete Combined with Ground Granulate Blast Furnace Slag and Macro Synthetic Fiber

**DOI:** 10.3390/ma17194735

**Published:** 2024-09-26

**Authors:** Shengzhao Cheng, Lisha Shen, Weige Chen, Haitang Zhu, Peibo You, Lu Chen

**Affiliations:** 1 China Construction Seventh Engineering Division Corp., Ltd., Zhengzhou 450002, China; chengshengzhao@aliyun.com (S.C.); chenlu@hotmail.co.uk (L.C.); 2 Yellow River Institute of Hydraulic Research, Zhengzhou 450003, China; 15638529115@163.com; 3 Henan Academy of Sciences, Zhengzhou 450046, China; 4 College of Civil Engineering, Henan University of Engineering, Zhengzhou 451191, China; htzhu@haue.edu.cn; 5 Department of Civil and Transportation Engineering, Henan University of Urban Construction, Pingdingshan 467036, China; 30070513@huuc.edu.cn

**Keywords:** building materials, fiber-reinforced concrete, macro synthetic fiber, ground granulated blast furnace slag, mechanical properties, chloride diffusion coefficient

## Abstract

Concrete with good mechanical properties and durability has always been a necessity in engineering. The addition of fibers and supplementary cementitious materials to concrete can enhance its mechanical and durability performance through a series of chemical and physical interactions. This study aims to investigate the effects of key parameters on the compressive strength, splitting tensile strength, and chloride penetration resistance of concrete combined with ground granulate blast furnace slag (GGBS) and macro polypropylene synthetic fiber (MSF). Based on the Taguchi method, a total of eighteen mixtures were evaluated, considering the effects of GGBS content, MSF content, water-to-binder (*w*/*b*) ratio, and chloride solution concentration on concrete properties. The results showed that the *w*/*b* ratio has a significant impact on the properties of concrete, which are enhanced by a decrease in *w*/*b* ratio. The GGBS content had little effect on the 28-day strength of concrete, which even decreased with a large GGBS content, but GGBS had a positive effect on the long-term strength of concrete. Moreover, the chloride penetration resistance of concrete was enhanced by an increase in the GGBS content. The MSF content had no obvious effects on the compressive strength and chloride penetration resistance of concrete, but it could enhance the splitting tensile strength to some extent, and this enhancement was more obvious over time. The chloride diffusion coefficient of concrete changed with the concentration of chloride solution, and the two increased simultaneously.

## 1. Introduction

Concrete is currently the most widely used artificial building material in the world. Due to its availability and low-cost advantages, it is extensively applied in various construction projects. Compared to other building materials such as steel, glass, wood, and brick, concrete not only has a lower price per kilogram but also has lower carbon emissions and energy consumption. Moreover, the use of concrete is enormous, estimated to exceed 10 billion cubic meters annually, playing an important role in economic development [1].

During its actual service, the durability of concrete structures is an unavoidable consideration, as they must face a variety of harsh environments. The intrusion of chloride ions is one of the main factors leading to the deterioration of concrete structures. Chloride ions are widely present in natural environments, particularly in marine environments, saline soils, and environments with de-icing salts [2,3]. Chloride ions penetrating the concrete reach the surface of the rebar, which destroys its passivation layer, causing rust expansion and leading to cracking and spalling of the concrete [4,5]. There are various strategies to enhance the resistance of concrete to chloride ion invasion, such as adding corrosion inhibitors to the concrete, coating surface, etc. However, the effectiveness of these methods is greatly reduced when the concrete cracks [6,7]. To ensure the long-term safe use of concrete structures, good mechanical properties and durability are required, especially the ability to protect the rebar from corrosion.

The use of fiber to reinforce concrete has attracted the attention of many scientists and building manufacturing institutions due to the benefits it provides. There are various types of reinforced fibers used in concrete, including steel fibers, glass fibers, natural fibers, and synthetic fibers. Steel fiber can greatly improve the tensile strength and bending strength of concrete, due to its extremely high strength and elastic modulus, and excellent bonding characteristics with concrete matrix [8,9]; however, the ability of steel fiber to maintain deactivation is questionable in the long-term harsh high-salt and high-humidity environment. Once steel fiber is seriously corroded, it will lead to the rapid degradation of the concrete structure’s performance [10]. Glass fiber has a good strengthening effect [11], but its alkali resistance is poor [12]. Natural fibers, such as sisal [13], palm [14], and sugarcane bagasse fibers [15], are cheap and easy to obtain, but their durability is poor [16]. Compared with the fibers of the above three materials, synthetic fiber has many advantages. Synthetic fibers are generally made of polymers; depending on the characteristics of the material, synthetic fibers can be elastic, flexible, or rigid [17]. Synthetic fibers made from materials such as polyethylene, polyvinyl alcohol, and polypropylene have been successfully used in the production of concrete [18]. Synthetic fiber can be obtained at low prices, resulting in labor and cost savings. Moreover, synthetic fiber has good durability and can exist stably in a concrete matrix. In addition, compared with steel fibers, synthetic fiber has multiple sustainable advantages, the most prominent of which is that it can reduce the carbon footprint, making it environmentally friendly. Some scholars have studied the influence of synthetic fiber on fresh concrete and hardened concrete, and the results show that the main advantages of synthetic fiber are the improvements in ductility in the post-crack region [19] and flexural toughness of concrete [20]. Moreover, the addition of synthetic fibers can change the failure mode of concrete, alter the essential characteristics of the brittle failure mode of concrete, and endow concrete with a certain degree of ductility. After the concrete cracks, the fibers between the cracks play a bridging role and transmit stress to the matrix on both sides of the cracks, thereby limiting the development of the cracks [21,22,23]. In addition, synthetic fiber can also effectively control plastic shrinkage cracking by improving the integrity of the fresh concrete [24]. These capabilities of synthetic fiber make it able to prevent water and pollutants from entering into deeper parts of a cracked concrete matrix, reducing the risk of reinforcement corrosion and deterioration of concrete structures. However, for uncracked concrete, the large number of fibers added into it will invariably introduce more interfaces between the fibers and cement matrix, thus affecting the overall permeability of the concrete; whether this will affect the ability of the concrete to be reinforced is worth further studying.

One of the characteristics of concrete with good durability is a large cement content, especially for high-strength and high-performance concrete. However, a high cement content also means greater consumption of natural resources, significant carbon dioxide emissions, and environmental pollution. Using supplementary cementitious materials (SCMs) is one of the effective ways to improve the environmentally friendly production of concrete [25]. Scientific and reasonable use of SCMs to replace ordinary Portland cement (OPC) can not only reduce the amount of cement in concrete and reduce the emission of greenhouse gases but also improve the characteristics of the concrete, such as reducing the hydration heat, enhancing its workability, and improving its durability. Ground granulated blast furnace slag (GGBS) is one of the commonly used SCMs and is a recycled industrial by-product in iron and steel manufacturing. Molten slag diverted from the iron blast furnace is rapidly chilled, producing glassy granules that yield desired reactive cementitious characteristics when ground into cement fitness. Using GGBS to replace part of OPC can reduce the heat required for hydration and also enable concrete to achieve faster strength development [26]. GGBS has been widely used in various projects to reduce the permeability and increase the durability of concrete. For example, 40% (mass percentage of cementitious materials) GGBS was used in the anchorage part of Japan’s Akashi Kaikyo Bridge [27]; 60% GGBS was adopted in the concrete caisson of Sydney Harbour Tunnel [28]; and 50% GGBS was utilized in the columns, decks, and barrier walls of Pinellas Bayway Bridge in Florida [26]. The addition of GGBS can improve the resistance of concrete to corrosive substances, but the difference in the activity between GGBS cement and OPC will inevitably affect the performance of concrete mechanical properties. Therefore, the influence of the proportional relationship between GGBS and OPC in concrete on the mechanical properties and durability of concrete deserves further study.

With the increasing application of concrete structures, high-quality concrete with good durability has become an urgent need in engineering practice. In this study, the Taguchi method was used to design combinations of the different influencing factors of concrete, and the compressive strength, splitting tensile strength, and chloride penetration resistance of each group were tested. The effects of water-to-binder ratio (*w*/*b*), GGBS content, and fiber content on the basic mechanical properties of concrete were studied. At the same time, the concentration of external immersed chloride solution was also considered in the study of the chloride penetration resistance of concrete. A range analysis and a variance analysis were used to study the influence and significance of each factor on the response. Based on the test results, mathematical models between the test variables and the responses were established by using a multiple regression analysis. Then, the signal-to-noise ratio (SNR) was introduced to analyze the influence of each factor on the concrete properties. In addition, scanning electron microscope (SEM) images and the failure mode morphology of concrete were also used to further illustrate the effects of the test variables on the responses. It is expected that the results of this study can provide a reference for the design of concrete combined with GGBS and fiber, so as to obtain concrete that achieves the basic mechanical properties and levels of durability.

## 2. Materials and Methods

### 2.1. Experimental Materials

**Figure 1:**
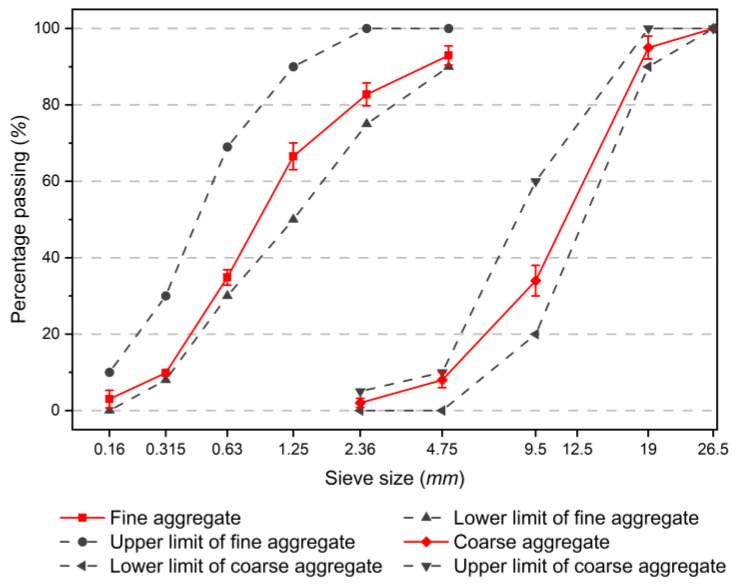
Sieve curves of fine and coarse aggregates.

The coarse aggregate used in this study is crushed limestone, and the fine aggregate is natural river sand with maximum particle sizes of 20 mm and 5 mm, respectively. The fineness modulus of river sand is 2.58, which can be classified as medium sand. Figure 1 shows the particle size distribution of coarse and fine aggregates. It can be seen that the grading of the aggregates is good, and the particle size curve complies with the provisions of ASTM C33 [29].

The OPC and GGBS used in this study were obtained from local sources. The grade of the OPC is 42.5, which can be approximately classified as Type I based on the ASTM C150 [30] standard, and the grade of the GGBS is S95, which can be classified as Grade 100 based on the ASTM C989 [31] standard. Table 1 and Table 2 are the physical and chemical properties of the OPC and GGBS, respectively.

The fiber used in this study is macro synthetic fiber (MSF) made of polypropylene; its properties are shown in Table 3. To ensure that the fresh concrete has good workability, a highly efficient polycarboxylate superplasticizer with a solid content of 40% was used.

### 2.2. Experiment Design

In this study, the *w*/*b*, GGBS content, and fiber content were used as influencing factors for the mechanical properties. At the same time, regarding the aspect of resistance to chloride penetration, the concentration of chloride solution was also considered as an influencing factor. Table 4 lists the factors and levels of experiment.

According to the factors and levels selected in this experiment, a mixed-level orthogonal table was used to design the experiment. Table 5 shows the arrangement of the orthogonal design, where A, B, C, and D represent the *w*/*b*, GGBS content, fiber content, and chloride solution concentration, respectively. Table 6 shows the corresponding concrete mix proportions.

### 2.3. Specimen Preparation and Testing

Specimens were prepared for the compressive strength, splitting tensile strength, and chloride penetration tests. First, the aggregate and fiber were added in a horizontal forced blender. After the blender was started, the cementitious materials and the water mixed with superplasticizer were added. Immediately after mixing, the mixtures were cast into a cubic specimen mold and compacted using a vibrating table. The specimens were then covered with a plastic film in the casting room for 24 h. Finally, the samples were demolded and cured in the curing room at 20 ± 2 °C and RH ≥ 95% until the test was performed.

#### 2.3.1. Mechanical Test

**Figure 2:**
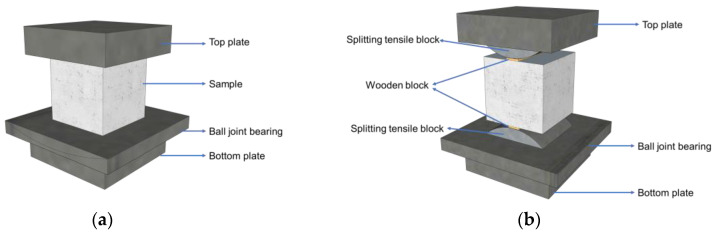
(**a**) The compressive strength test and (**b**) splitting tensile strength test of concrete.

Compressive strength and splitting tensile strength are basic mechanical properties of concrete. As shown in Figure 2, tests of these properties were conducted using a compression testing machine, the test process referenced the Chinese standard test methods for fiber-reinforced concrete (CECS 13-2009) [32]. The specimen size was 100 mm × 100 mm × 100 mm, three in each group, and the loading rate was 0.5 MPa/s for compressive strength and 0.05 MPa/s for splitting tensile strength. The strength was calculated using Equations (1) and (2):

(1) $$R_{CS}=\frac{P}{A_{1}}$$

(2) $$R_{TS}=\frac{2P}{\pi A_{2}}$$

where $R_{CS}$ is compressive strength (MPa); P is the maximum load at failure (N); and $A_{1}$ is the compression and splitting tensile areas, both of which are 100 ${mm}^{2}$. In this study, the substitution of GGBS for Portland cement reached 50% in some groups, and the large amount of SCMs slowed down the growth in the concrete strength. Therefore, in the strength test, not only the strength of the specimens at 28 days of standard age but also the long-term stable strength of the specimens after one year, that is 365 days, were tested.

#### 2.3.2. Chloride Penetration Test

##### Wetting–Drying Cycles

The chloride penetration test referenced AASHOT T259 [33] and NT Build 443-94 [34]. Several pretreatments were carried out on the specimens before the wetting–drying cycles. In order to ensure the chloride penetration in only the one-dimensional direction, besides the one side selected as the exposed surface, the other five sides were coated with epoxy resin. Then, all specimens were put into a chest for wetting–drying cycles. A salinometer was used to ensure that the sodium chloride solution concentration error was within 0.2%. The experiment lasted for 24 cycles (2 days wet and 2 days dry for one cycle). This test was completed indoors, and the daily average temperature and relative humidity during the test were 23.6 °C and 67.8%, respectively.

##### Determination of Chloride Content

**Figure 3:**
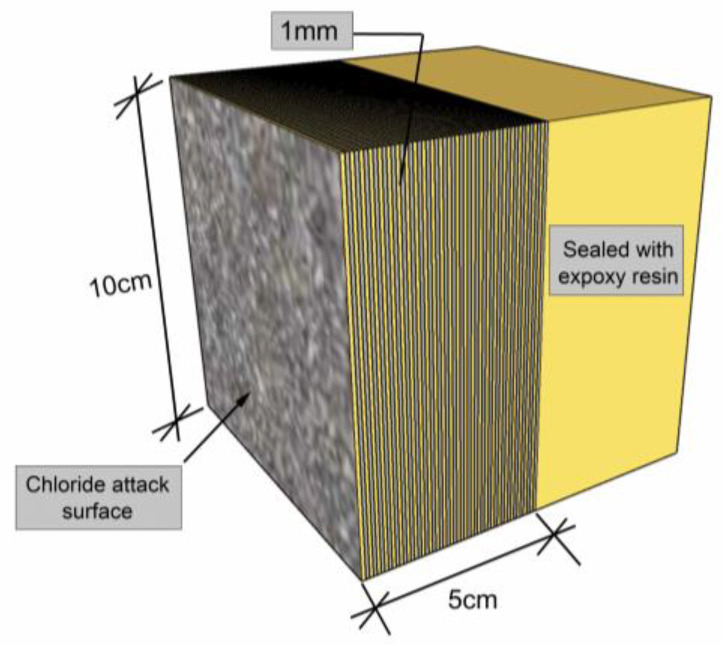
Schematic diagram of the grinding process of the concrete specimen.

After 24 wetting–drying cycles, all specimens were taken out of the salt solution with different concentrations. After these specimens were dried in the natural environment, they were clamped on a concrete mill for grinding. Figure 3 shows a schematic diagram of the grinding process of the concrete specimen. From the chloride attack surface, the concrete was milled layer by layer with a constant thickness of 1 mm, and the concrete powder samples were collected into plastic sealing bags. Through this procedure, more data points can be provided in the process of establishing the chloride profile to make the results more accurate. All powder samples were then dried in an electric blast dryer, and coarse particles were removed using a No.100 sieve (# 0.15 mm). An automatic potentiometric titrator was used to determine the acid-soluble chloride in concrete; the titration solution was 0.01 mol/L silver nitrate solution, and the titrator automatically recorded the consumed volume of this solution. The chloride percent by mass of concrete was calculated by using

(3) $$P=\frac{0.3545V}{1000m}\times 100\%$$

where P is the mass percentage of chloride in the sample (%), V is the volume of consumed silver nitrate solution (mL), and m is the mass of the sample (g).

##### Chloride Diffusion Coefficient

The main mechanism of chloride transport in saturated concrete is diffusion, which is driven by the ion concentration gradient inside and outside the concrete. Fick’s law, as shown in Equation (4), is often used to describe the diffusion of chloride in concrete. Furthermore, when the latter is considered to be one-dimensional in a semi-infinite medium, an error equation solution of Fick’s second law is often used to describe the experimental data of the chloride profile in concrete, as shown in Equation (5):

(4) $$\frac{\partial C}{\partial t}=D\frac{\partial^{2}C}{\partial x^{2}}$$

(5) $$C\left(x,t\right)={C_{0}+(C}_{s}-C_{0})\left[1-erf\left(\frac{x}{2\sqrt{D_{app}\cdot t}}\right)\right]$$

where x is the depth from the chloride attack surface (m), t is the exposure time (s), $C\left(x,t\right)$ is the chloride content (% by mass of concrete) at depth x and time t, $C_{0}$ is the initial chloride content (% by mass of concrete), $C_{s}$ is the chloride content at the surface (% by mass of concrete), $D_{app}$ is the apparent chloride diffusion coefficient ($m^{2}/s$), and erf is the statistical error function.

It is worth mentioning that Fick’s second law can only be used in the diffusion zone of chloride in concrete. However, for concrete subjected to wetting–drying cycles, the chloride transport in concrete involves both diffusion and convection, in this case, the chloride profile does not always follow Fick’s law, and the region that involves both mechanisms is often called the convection zone [35]; it is usually the area from the exposed surface of the concrete to the point of peak chloride concentration. Its depth is a key parameter that can affect the prediction of the chloride profile in concrete cover, especially when using Fick’s second law to calculate the chloride content. Due to the presence of the convection zone, using the traditional Fick’s second law (i.e., Equation (5)) to calculate the diffusion coefficient of chloride can lead to distorted results. To settle this defect [36], the usual procedure is to move the axis (x=0) to the end of the convection zone where the chloride content reached its maximum, replacing the surface chloride content ($C_{s}$) with the maximum chloride content ($C_{m}$) and using $\left(x-x_{c}\right)$ to replace x, where $x_{c}$ is the depth of the convection zone. Therefore, the chloride profile in concrete containing a convection zone can be described as follows:

(6) $$C\left(x,t\right)=C_{0}+{(C}_{m}-C_{0})\left\{1-erf\left[\frac{x-x_{0}}{2\sqrt{D_{app}t}}\right]\right\}$$

## 3. Results and Discussion

### 3.1. Experimental Results for 28-Day and 365-Day Compressive Strength

Table 7 shows the test results for 28-day compressive strength and 365-day compressive strength. Obviously, the 365-day compressive strength of all groups was higher than their 28-day compressive strengths. The mean value of the growth coefficient is 1.15, the standard deviation is 0.108, and the coefficient of variation is 9.4%. The results showed that after 365 days of curing, the compressive strength of concrete increased by an average of 15% compared to only 28 days of curing.

A range analysis is an analytical approach using a Taguchi array. A range, which represents the influence of different factors on the test results, with a larger value indicates that the change in each level of factors in the test range will lead to greater changes in the response [37]. According to the range analysis, the order of influence of each factor on the test results can be deduced by comparing the range of each factor.

Table 8 presents the range analysis results for 28-day and 365-day compressive strength. It can be found that the range of specimens cured for 28 or 365 days had the following order: $R_{A}>R_{B}>R_{C}$. Therefore, the influence of each factor on the compressive strength from large to small is w/b, GGBS content, and fiber content.

A range analysis cannot distinguish the data fluctuation caused by test errors and a change in test conditions. In order to remedy the defects of the range analysis, a repeated analysis of variance (ANOVA) test without any empty columns was adopted. Table 9 and Table 10 show the ANOVA results for 28-day and 365-day compressive strength, respectively. As shown in Table 9, the w/b had an extremely significant effect, with the highest contribution rate of 79.38% to the variance, while the contribution rate of GGBS content was small (5.24%) and the influence of fiber was small, which was within the error. Similarly, for the 365-day long-term compressive strength, each factor had the same influence regular pattern. The contribution rates of w/b and GGBS content were 59.02% and 2.86%, respectively.

Based on the experimental results, mathematical models for 28-day and 365-day compressive strength were developed using a multiple regression analysis. Considering the consistency of the order of magnitude of the regression coefficients, the independent variable, fiber content, was substituted by volume fraction ($V_{f}$); 3 $kg/m^{3}$ fiber content corresponds to 0.33% $V_{f}$, 6$\;kg/m^{3}$ fiber content corresponds to 0.66% $V_{f}$, and 9$\;kg/m^{3}$ fiber content corresponds to 0.99% $V_{f}$.

The mathematical model for 28-day compressive strength is as follows:

(7) $$y=73.21-63.16x_{1}-26x_{2}^{2}+8.86x_{2}-1.23x_{3}$$

$$R^{2}=0.8422$$

where y is the 28-day compressive strength, $x_{1}$ is the w/b, $x_{2}$ is the GGBS-to-cement ratio, and $x_{3}$ is the fiber volume fraction (%).

And, the mathematical model for 365-day compressive strength is as follows:

(8) $$y=85.99-82.12x_{1}+17.76x_{2}-4.55x_{3}$$

$$R^{2}=0.7803$$

where y is the 365-day compressive strength, $x_{1}$ is the w/b, $x_{2}$ is the GGBS-to-cement ratio, and $x_{3}$ is the fiber volume fraction (%).

**Figure 4:**
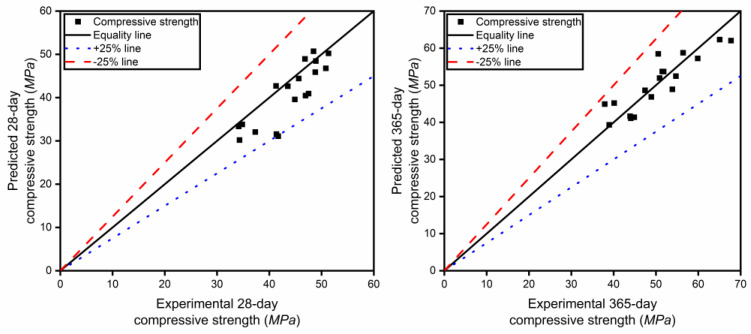
Comparison of the compressive strength between model calculation and experimental data.

In Figure 4, the experimental values and the predicted values, which were calculated using the proposed mathematical model, for the 28-day and 365-day compressive strength were compared. It was found that the data points are basically distributed in the range of ±25% from the mean line, indicating that the mathematical model can be used to predict the compressive strength value.

### 3.2. Experimental Results for 28-Day and 365-Day Splitting Tensile Strength

Table 11 shows the results of 28-day and 365-day splitting tensile strength. It was found that the 365-day splitting tensile strength in most groups is higher than the 28-day strength. The mean value of the strength growth coefficient is 1.12, the standard deviation is 0.178, and the coefficient of variation is 15.9%. The results also indicated that the splitting tensile strength shows an increasing trend over time. In addition, the variation in the splitting tensile strength growth coefficient was greater than that of the compressive strength growth coefficient, which shows that the dispersion degree of splitting tensile strength is higher than that of compressive strength.

Table 12 is the range analysis results of 28-day and 365-day splitting tensile strength. It can be found that the range order is the same for the specimens cured for 28 days and 365 days: $R_{A}>R_{B}>R_{C}$. Therefore, the influence of each factor on the splitting tensile strength from large to small is w/b, GGBS content, and fiber content. It is worth mentioning that the range of each factor at 365 days had different degrees of improvement compared with at 28 days, but the fiber content range showed the largest increase, from 0.106 to 0.413, with an increase of 289.6%. This also showed that the role of fibers in long-term aging was more obvious.

Table 13 and Table 14 presented the ANOVA for 28-day and 365-day splitting tensile strength, respectively. The ANOVA results of splitting tensile strength for 28 days showed that, compared with GGBS content and fiber content, w/b had an extremely significant influence. Its contribution to the variance is 49.72%, whereas other factors could be within the error. For the 365-day long-term splitting tensile strength, the influence of w/b was significantly decreased, but the contribution rate of w/b was still the highest of all the factors, at 15.72%. Although the contribution rate of fiber content was only 0.08%, it could be distinguished from the error, which further showed that the effect of fiber content on the splitting tensile strength of concrete is more obvious in the long term.

Based on the experimental results, mathematical models for 28-day and 365-day splitting tensile strength were proposed. The regression equation for 28-day splitting tensile strength is as follows:

(9) $$y=1.47-20.87x_{1}^{2}+15.4x_{1}+2.05x_{2}^{2}-1.81x_{2}+0.16x_{3}$$

$$R^{2}=0.7951$$

where y is the 28-day splitting tensile strength; $x_{1}$ is w/b; $x_{2}$ is the GGBS-to-cement ratio; and $x_{3}$ is the fiber volume fraction (%).

And, the regression equation for 365-day splitting tensile strength is as follows:

(10) $$y=8.76+15.03x_{1}^{2}-17.37x_{1}-5.80x_{2}^{2}+3.22x_{2}+1.27x_{3}^{2}-1.05x_{3}$$

$$R^{2}=0.7678$$

where y is the 365-day splitting tensile strength, $x_{1}$ is w/b, $x_{2}$ is the GGBS-to-cement ratio, and $x_{3}$ is the fiber volume fraction (%).

**Figure 5:**
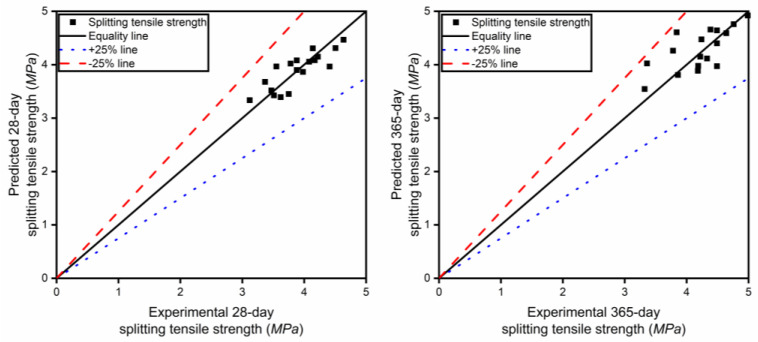
Comparison of the splitting tensile strength between model calculation and experimental data.

As for the analysis method for compressive strength, the experimental values and the predicted values, which were calculated using the proposed equation, for the 28-day and 365-day splitting tensile strengths were also compared and graphically presented in Figure 5. It can be seen from the comparison diagram that the proposed equation is suitable for predicting the splitting tensile strength.

### 3.3. Experimental Results for Chloride Penetration Resistance

**Figure 6:**
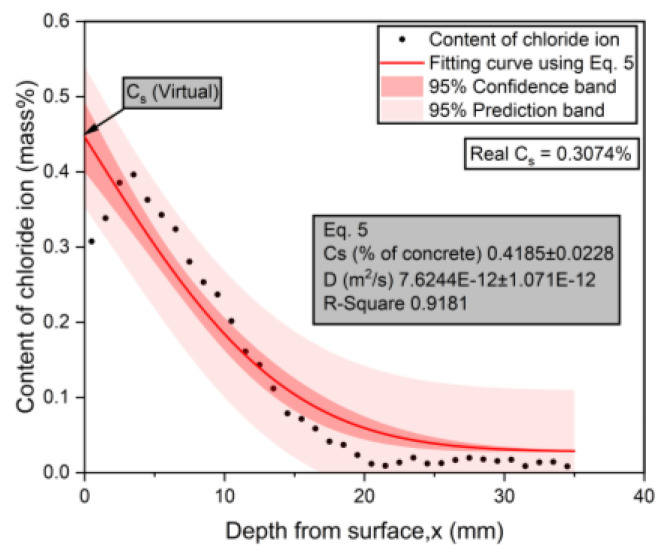
The calculation results of $D_{app}$ using Equation (5).

**Figure 7:**
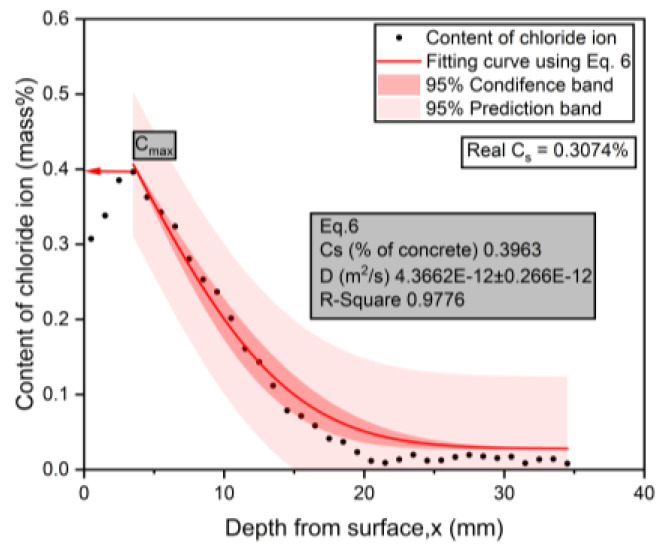
The calculation results of $D_{app}$ using Equation (6).

The typical chloride profile measured during the test for group 13 is presented in Figure 6 and Figure 7. The data points were fitted using Equations (5) and (6), respectively, and the chloride diffusion coefficients were also calculated. It can be found that due to the presence of convection zones, when Equation (5) was used, the calculated surface chloride ion concentration was much higher than the actual surface chloride concentration, while Equation (6) ignored the convection zone in the calculation and directly calculated the peak chloride concentration as the equivalent surface chloride concentration. A comparison of the two sets of results showed that the R-square value from Equation (6) is larger than that from Equation (5), indicating that Equation (6) has a better fitting degree for the chloride profile. The surface chloride concentration, $C_{s}$, obtained by Equation (5) was larger than the actual $C_{s}$ ($C_{s,calculated}=0.4185\%>C_{s,actual}=0.3074\%$). This result indicates that in the process of chloride transport in the surface layer of concrete, the concentration gradient is the main driving force, which is inconsistent with the actual chloride profile. Because the $C_{s}$ value from Equation (5) was higher, the predicted service life to reach the chloride threshold will be shorter than that when using the value from Equation (6). Therefore, this equation is better at describing the law of chloride penetration in concrete under wetting–drying cycles.

After 24 wetting–drying cycles, the apparent chloride diffusion coefficients ($D_{app}$) of the specimens were calculated using Equation (6), and the results are represented in Table 15.

Table 16 shows the range analysis results of $D_{app}$ after 24 wetting–wetting cycles. It can be found that the range order is $R_{A}>R_{B}>R_{D}>R_{C}$. In other words, the factors in the chloride penetration resistance of concrete had the following order of importance: w/b, GGBS content, chloride solution concentration, and fiber content.

Table 17 shows the ANOVA results of $D_{app}$ after 24 wetting–drying cycles. It showed that, compared with other factors, w/b has a remarkable impact, and the contribution rate of variance is 74.32%. This was followed by the GGBS content and chloride solution concentration, which had contribution rates of 7.45% and 3.48%, respectively. The influence of fiber content on chloride penetration resistance of concrete is not apparent, and it can be within the error.

Based on the experimental results, mathematical models for $D_{app}$ were proposed, and the regression Equation is as follows:

(11) $$y=-11.64+36.85x_{1}-6.55x_{2}+1.25x_{3}+0.5x_{4}$$

$$R^{2}=0.9205$$

where y is the $D_{app}$ of chloride ions after 24 drying–wetting cycles, $x_{1}$ is the w/b, $x_{2}$ is the GGBS-to-cement ratio, $x_{3}$ is the fiber volume fraction (%), and $x_{4}$ is the solution concentration of chloride (%).

**Figure 8:**
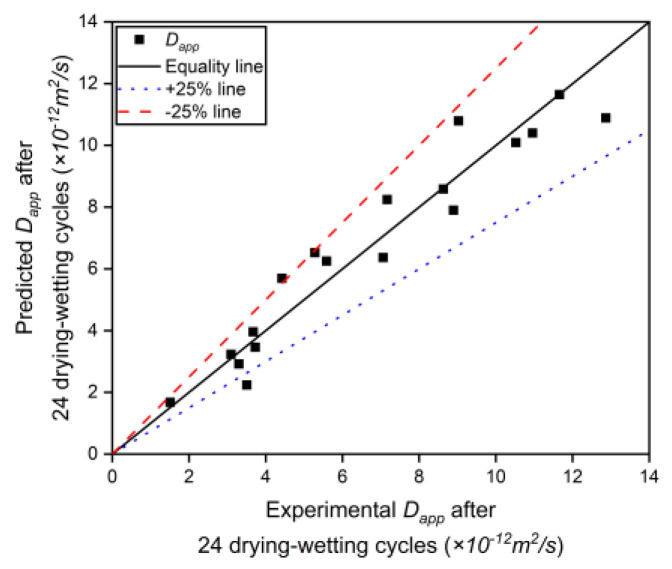
Comparison of the $D_{app}$ between model calculation and experimental data.

Figure 8 shows the comparison of the experimental values and predicted values of $D_{app}$, and there is good consistency between both sides.

### 3.4. Signal-to-Noise Ratio (SNR)

In the Taguchi orthogonal array, rows and columns represent the number of factors and experiments, respectively, and the influence of factors on the response was determined by the symmetric change in factor level. SNR was utilized to further analyze the results. The value of SNR represents the variance around a specific value, in other words, the changes in the response of several tests to an indicator. The calculation of SNR can be divided into three categories:

(12) $$\mathrm{Smaller}\;\mathrm{the}\;\mathrm{better}\;\frac{S}{N}=-10{log}_{10}\left[\frac{1}{n}\sum_{i=1}^{n}y_{i}^{2}\right]$$

(13) $$\mathrm{Lager}\;\mathrm{the}\;\mathrm{better}\;\frac{S}{N}=-10{log}_{10}\left[\frac{1}{n}\sum_{i=1}^{n}\frac{1}{y_{i}^{2}}\right]$$

(14) $$\mathrm{Nominal}\;\mathrm{the}\;\mathrm{better}\;\frac{S}{N}=-10{log}_{10}\left[\sigma^{2}\right]$$

where n is the number of test repetitions and $y_{i}$ is the experimental obtained value [38].

SNR reflects the influence of control factors on the response, and a higher SNR value means a better response value. In this paper, for the “the larger the better” condition, Equation (13) was selected to calculate the SNR values for the compressive strength and splitting tensile strength of concrete, while for the “the smaller the better” condition, Equation (12) was selected for the apparent chloride diffusion coefficient. Table 18 lists the SNR values of each index for each group.

#### 3.4.1. SNR Results of Compressive Strength

**Figure 9:**
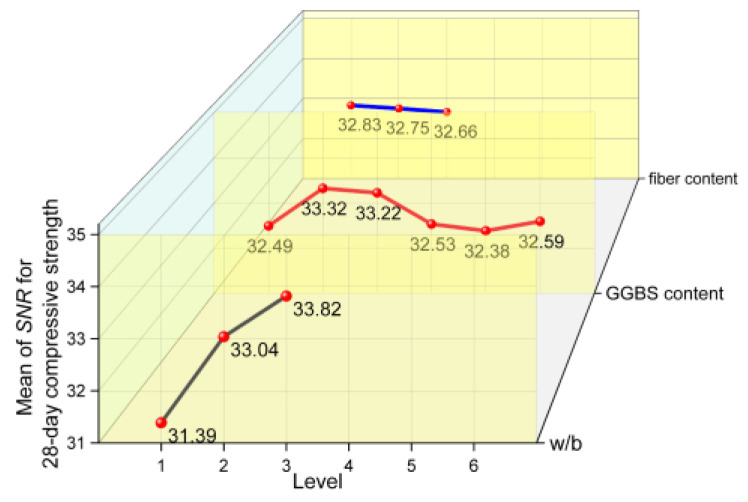
The mean main effects plots of SNR for 28-day compressive strength.

**Figure 10:**
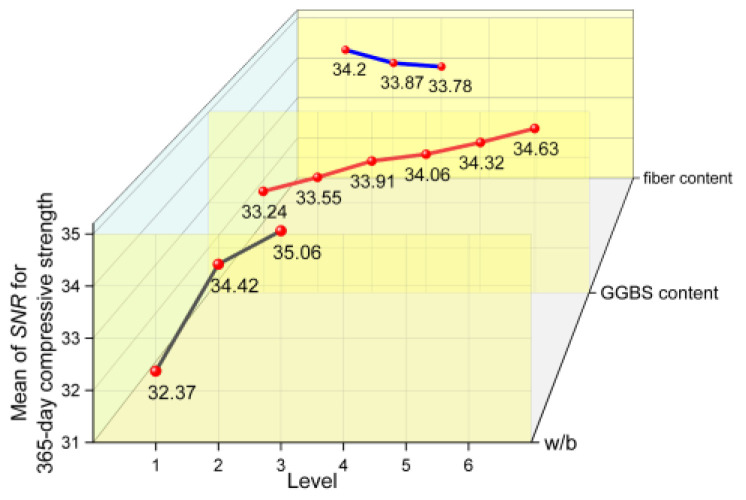
The mean main effects plots of SNR for 365-day compressive strength.

In Figure 9 and Figure 10, it can be observed that the compressive strength decreases with an increase in w/b, regardless of whether the specimen was cured for 28 days or 365 days, and the compressive strength reaches the maximum when w/b is 0.36. In general, a decrease in the w/b decreases the porosity of cementitious matrix, upgrades the microstructure of the interfacial transition zone around the aggregate, and increases the density and strength of concrete.

The effect of GGBS content on the compressive strength at 28 days and 365 days are quite different. For a short age of 28 days, when the GGBS content is low (10% and 20%), the compressive strength shows a slight increase, and with a further increase in GGBS content, the compressive strength shows a decreasing trend. However, even with the highest content (50%) considered in this study, the compressive strength of concrete is not significantly reduced. For the extended age of 365 days, it can be found that the compressive strength increases with the GGBS content. Compared with OPC, the GGBS used in this study had a larger specific surface area and smaller particle size. In the early stages, GGBS could have a certain physical filling effect, which would improve the compactness of the hardened cement slurry. The GGBS also provided large nucleation surfaces for hydration products and promoted their formation [39]. However, with a further increase in GGBS content, the OPC content obviously decreased, which led to a decrease in the hydration products. As the hydration process continued, the GGBS activity in the alkaline environment was further stimulated, and a secondary hydration reaction occurred, which consumed calcium hydroxide (CH) and formed a dense hydrated calcium silicate (C−S−H) gel [40]. Thus, the concrete had a higher strength.

**Figure 11:**
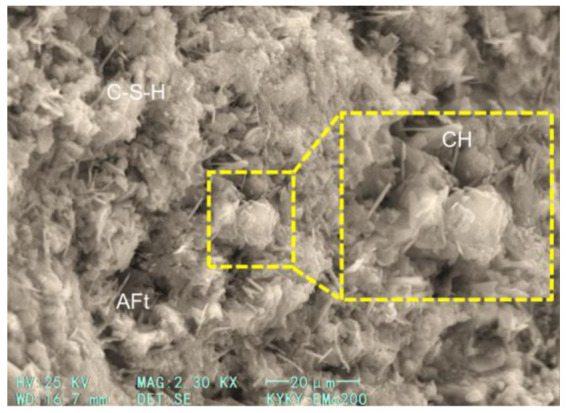
SEM image of hardened concrete at 28 days.

**Figure 12:**
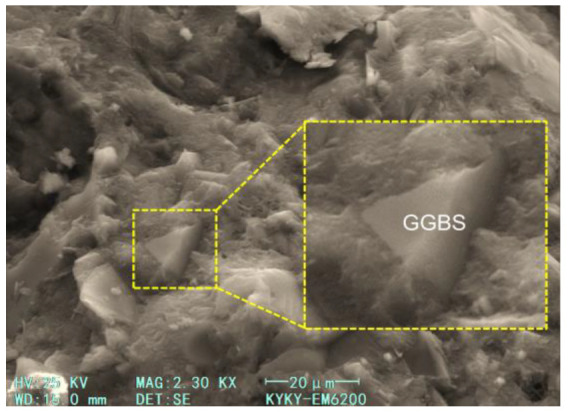
SEM image of hardened concrete at 365 days.

The micro morphology of hardened pastes can be used to characterize the compactness of the paste structure, the morphological characteristics of the hydration products, and the participation degree of cementitious material particles in hydration. It is of great significance to analyze the hydration process and reveal the hydration mechanism. Figure 11 and Figure 12 show the SEM images of the 28-day and 365-day freshly fractured surfaces of the samples, respectively. In Figure 11, it can be seen that the hydration products of the samples (the specimens of group 17) were abundant at 28 days, with these hydration products distributed on the surfaces of the unhydrated particles. The paste structure was not dense enough, and the bonding between hydration products was not compact. Laminated CH and a large number of flocculent C−S−H gel can also be observed in the figure, and needle ettringite (AFt) can be found in large pores. The micromorphological appearance of the sample can be seen in the SEM image of the 365-day specimen (Figure 12); sufficient hydration products were produced with an increase in age, and the matrix became dense. Because the latent reactivity of the GGBS was excited, the degree of GGBS reaction was increased. It can be observed that the polygonal GGBS particles were surrounded by a dense gel and tightly adhered to the surrounding matrix, which filled the pores well, and the hardened paste was compact. Therefore, at the early age of 28 days, the lower GGBS content could improve the strength of the concrete, whereas at the greater age of 365 days, a larger GGBS content could also effectively improve the strength.

It also can be found in Figure 9 and Figure 10 that the 28-day and 365-day compressive strengths decreased slightly with the increases in fiber content, but the overall effect was not significant. This may be because the elastic modulus of MSF used is far smaller than that of the concrete matrix. In general, the elastic modulus of concrete with ordinary strength is 25~35 GPa, while the elastic modulus of MSF is only 3.5 GPa. In a sense, dispersing the fibers in the concrete was equivalent to introducing a large number of randomly distributed low modulus defects [41]. When the specimens were subjected to an external load, an internal stress concentration could easily occur, which decreased the compressive strength of concrete.

#### 3.4.2. SNR Results of Splitting Tensile Strength

**Figure 13:**
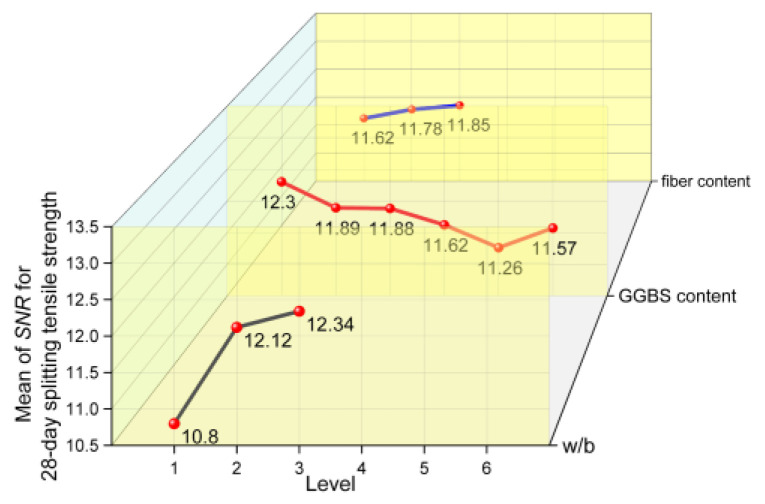
The mean main effects plots of SNR for 28-day splitting tensile strength.

**Figure 14:**
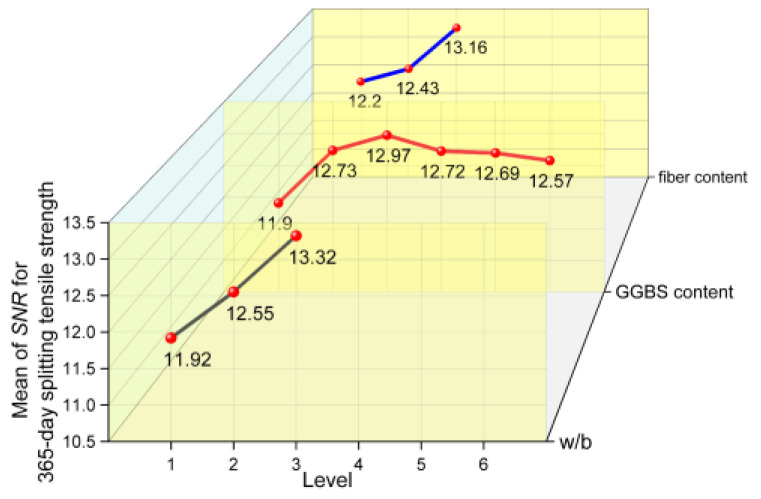
The mean main effects plots of SNR for 365-day splitting tensile strength.

The splitting tensile strength is an indirect index reflecting the tensile performance and toughness of concrete. The SNRs of 28-day and 365-day splitting tensile strength are plotted in Figure 13 and Figure 14, which shows the variation in splitting tensile strength at each factor level. It can be observed from the figure that the variation law of splitting tensile strength with a change in w/b is the same as that of compressive strength, where both decrease with an increase in w/b. The 28-day splitting tensile strength decreases with the increase in the GGBS content. And, compared with the concrete without GGBS, the 365-day splitting tensile strength of concrete has different degrees of growth with a change in the GGBS content. The effects of w/b and GGBS content on the splitting tensile strength of concrete mechanisms are similar to those for the compressive strength. However, in contrast to the effect of fiber content on the compressive strength, the splitting tensile strength of concrete increases with an increase in fiber content, and the range of increase for 365 days is greater than that for 28 days. In other words, the strengthening effect of fiber on the splitting tensile strength of concrete is more obvious after a long curing period.

**Figure 15:**
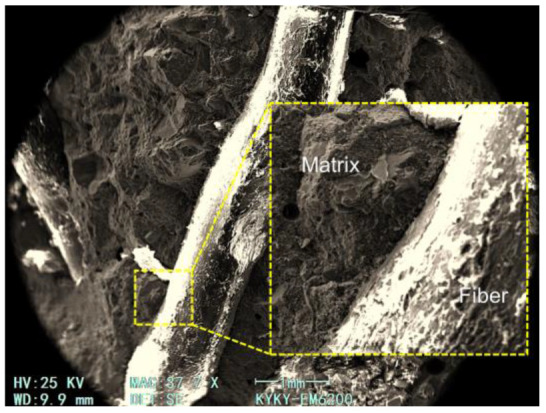
SEM image of MSF in concrete.

**Figure 16:**
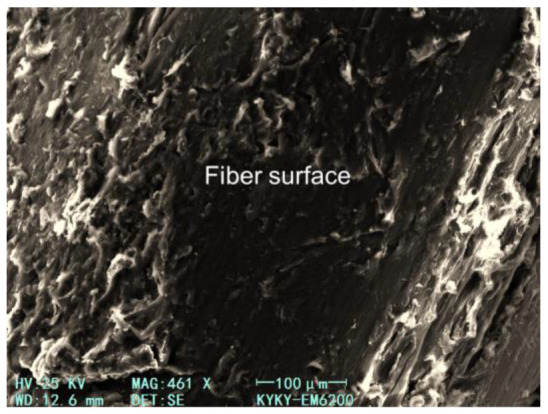
SEM image of MSF surface after debonding from concrete matrix.

According to fiber spacing theory, the theoretical calculation of the MSF spacing used in this study is 10~20 mm [42], which matched the size of coarse aggregates. Thus, the fiber could be evenly dispersed in concrete. The SEM images of MSF presented in Figure 15 and Figure 16 show that the fiber has a fairly good bonding state in the concrete, and the crude surface of the fiber further strengthened the bond between the fiber and the concrete matrix.

**Figure 17:**
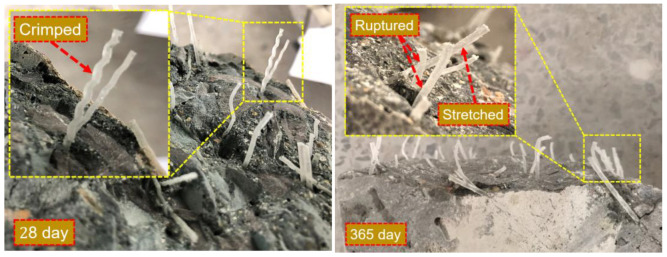
The morphology of the fiber in concrete during the 28 day and 365 day splitting tensile tests.

In addition, the effect of fiber on splitting tensile strength was also reflected in the surface morphology of the fiber in the concrete failure sections. Figure 17 shows the pull-out states of the fiber in the concrete sections after the splitting tensile strength test after curing for 28 days and 365 days. During the splitting tensile test, with an increase in external load, the microcracks in the weak interface of the matrix penetrated through, and the fiber bridged in the fracture surface began to bear the load and transferred it to the uncracked part. As the cracks continued to open, the bridging fibers were constantly pulled out or broke. The pull-out resistance of the fiber was mainly determined by the bonding between the fiber and the matrix and the mechanical interaction caused by the special shape of the fiber surface [43]. It can be seen from the 28-day concrete failure morphology that after the section was completely fractured, some fibers still showed a crimped pattern on the surface, which indicates that the bond between the fiber and the matrix was weak and the effect of the fiber on the splitting tensile strength was not obvious. From the fracture morphology of the 365-day concrete, it can be seen that most of the fiber was stretched and some fibers even ruptured. This phenomenon indicated that the interface between fiber and concrete matrix increased with the increase in age. Therefore, after a long period of curing, the fiber played a greater role in improving the tensile strength of the concrete.

#### 3.4.3. SNR Results of Apparent Chloride Diffusion Coefficient

**Figure 18:**
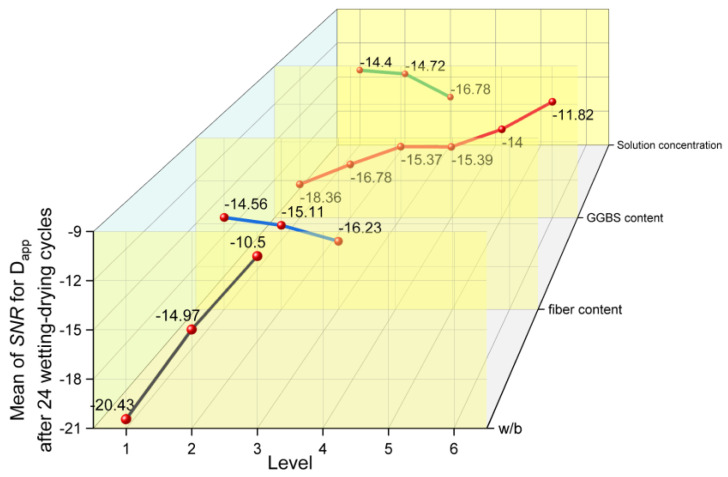
The main effects plots of SNR for $D_{app}$.

Figure 18 shows the influences of several factors and levels on the $D_{app}$ after 24 wetting–drying cycles. It can be found that the chloride penetration resistance of concrete increases with a decrease in w/b; it also increased with an increase in the GGBS content and slightly decreased with the increase in the fiber content and external chloride solution concentration.

A decrease in w/b increased the density of concrete and enhanced its ability to resist chloride penetration. The addition of GGBS could also make the concrete more impenetrable, block the transport path of chlorides, and significantly change the hydration products. In addition to the C−S−H gel generated by the secondary hydration of GGBS, Friedel’s salt also formed in the concrete due to the higher alumina content of GGBS. On the whole, the concrete mixed with GGBS can absorb more chlorides, which enhanced its chloride penetration resistance [44]. The addition of fiber introduced more interfaces in concrete, which affected its overall anti-chloride penetration performance [45,46]. The interface increased with an increase in fiber content, and the chloride penetration resistance of concrete exhibited a slight weakening trend. Chloride transport in concrete is a complicated process [47], including capillary suction, migration in an electrical field, and wick action, and the main driving force of chloride transport depends on the concentration gradient between the internal and external parts of the concrete, with a faster transmission speed of chlorides at a high concentration gradient. Therefore, the chloride penetration resistance of concrete decreased with an increase in the external solution concentration. And, this also indicated that the chloride penetration resistance of concrete is related not only to the properties of the concrete itself but also to the external conditions encountered by the concrete.

Therefore, when considering the basic mechanical properties and chloride penetration resistance of concrete, w/b is the most important control factor in the design of the ordinary strength of concrete, and a lower w/b should be selected within the required strength range. Second, under the condition of meeting the strength requirements, a large amount of GGBS can be used to reduce the amount of OPC, which will improve the utilization rate of high-energy-consumption cement and achieve the best use of the materials. Then, the addition of MSF can improve the tensile strength of concrete. However, it has no obvious effect on its compressive strength and chloride penetration resistance; therefore, the appropriate dosage of MSF can be selected in consideration of the cost.

## 4. Conclusions

In this study, several parameters that were expected to influence the basic mechanical properties and chloride penetration resistance of concrete were considered. The Taguchi method was adopted in the group design, and the SNR was introduced to analyze the influence law of various parameters on the characteristics of concrete. The following are the main conclusions drawn from this study:

1. w/b had a significant impact on the compressive strength, splitting tensile strength, and chloride penetration resistance of concrete, with these properties of concrete all improved or enhanced with a decrease in w/b.
2. The addition of GGBS could improve the long-term strength and chloride penetration resistance of concrete. A lower GGBS content (10%~20%) could slightly enhance the 28-day compressive strength of concrete, but with a further increase in the GGBS content (30%~50%), the compressive strength of concrete decreased. However, the 365-day compressive strength of concrete increased with an increase in the GGBS content. Compared with the OPC concrete, adding GGBS could reduce the 28-day splitting tensile strength of concrete. However, similar to the compressive strength, the addition of GGBS played a positive role in the 365-day splitting tensile strength. And, GGBS could improve the ability of concrete to resist chloride penetration. Within the range of GGBS content considered in this study, the chloride penetration resistance of concrete increased with an increase in GGBS content.
3. The addition of MSF has no obvious effect on the compressive strength and chloride penetration resistance of concrete. MSF could improve the splitting tensile strength of concrete, and the strengthening effect after 365 days was more obvious than that after 28 days.
4. $D_{app}$ increased with an increase in the concentration of chloride solution used for wetting–drying cycles, which indicated that the ability of concrete to resist chloride penetration is related not only to the properties of the concrete itself but also to the external environment encountered by the concrete.
5. Based on the considered parameters, the mathematical regression models of compressive strength, splitting tensile strength, and chloride permeability of concrete were established. There is good consistency between the calculated and measured values of the model, but the accuracy of the model needs to be further improved.

In conclusion, the addition of MSF and GGBS can enhance the performance of concrete, but it can also cause some negative effects. This study only conducted preliminary research on the basic mechanical properties and resistance to chloride penetration under drying–wetting cycles. Future research can be combined with specific application backgrounds to further study the more complex mechanical properties of concrete containing MSF and GGBS, including but not limited to bending, direct tension, impact, etc. In terms of durability, in addition to chloride ion penetration, resistance to sulfate corrosion, freeze–thaw cycles, and high temperature resistance are all worthy of further research.

## Figures and Tables

**Table 1:** Physical properties of the OPC and GGBS.

Physical Properties	OPC	GGBS
$\mathrm{Specific}\;\mathrm{surface}\;\mathrm{area}\;(m^{2}/kg$)	355	432
$\mathrm{Bulk}\;\mathrm{density}\;(g/m^{3}$)	3.10	2.89
Initial setting time (min)	225	-
Final setting time (min)	277	-
3-day compressive strength (MPa)	32.4	-
3-day tensile strength (MPa)	6.2	-

**Table 2:** Chemical composition of the OPC and GGBS.

Chemical Composition	OPC (%)	GGBS (%)
${SiO}_{2}$	20.60	32.91
${Al}_{2}O_{2}$	4.81	15.36
${Fe}_{2}O_{3}$	3.83	0.74
CaO	63.78	37.11
MgO	1.58	8.52
${SO}_{3}$	2.30	0.18
${Cl}^{-}$	0.03	0.018
Heat loss	2.81	0.12

**Table 3:** Properties of the MSF.

Length (mm)	Diameter (μm)	Density (g/cm^3^)	Tensile Strength (N/mm^2^)	Elasticity Modulus (GPa)	Picture of the Fibers
30	800	0.91	450	3.5	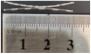

**Table 4:** Factors and levels of experiment.

Factors	Levels					
	1	2	3	4	5	6
A. w/b	0.55	0.44	0.36			
B. GGBS content (%mass of cementitious material)	0	10	20	30	40	50
$C.\;\mathrm{Fiber}\;\mathrm{content}\;(kg/m^{3}$)	3	6	9			
D. Sodium chloride solution concentration (%)	3.5	5.0	7.0			

**Table 5:** The mixed-level orthogonal array for the Taguchi design.

Exp. No.	Experimental Array L18			
	A	B	C	D
1	1	1	1	1
2	2	1	2	2
3	3	1	3	3
4	1	2	1	2
5	2	2	2	3
6	3	2	3	1
7	1	3	2	3
8	2	3	3	1
9	3	3	1	2
10	1	4	3	2
11	2	4	1	3
12	3	4	2	1
13	1	5	2	1
14	2	5	3	2
15	3	5	1	3
16	1	6	3	3
17	2	6	1	1
18	3	6	2	2

Note: Factor D, the concentration of sodium chloride solution for wetting–drying cycles, was only considered in the design of the chloride penetration test, and it was used as an error column in the design of the basic mechanical property test. Exp. No. is the abbreviation for experiment number.

**Table 6:** Mix proportions (kg/m^3^) for each group.

Exp. No.	OPC	GGBS	Water	Fine Aggregate	Coarse Aggregate	MSF	Superplasticizer
1	340	0	170	756.0	1134.0	3	1.70
2	400	0	160	680.8	1159.2	6	2.00
3	500	0	150	612.5	1137.5	9	2.50
4	306	34	170	756.0	1134.0	3	1.53
5	360	40	160	680.8	1159.2	6	1.80
6	450	50	150	612.5	1137.5	9	2.25
7	272	68	170	756.0	1134.0	6	1.36
8	320	80	160	680.8	1159.2	9	1.60
9	400	100	150	612.5	1137.5	3	2.00
10	238	102	170	756.0	1134.0	9	1.19
11	280	120	160	680.8	1159.2	3	1.40
12	350	150	150	612.5	1137.5	6	1.75
13	204	136	170	756.0	1134.0	6	1.02
14	240	160	160	680.8	1159.2	9	1.20
15	300	200	150	612.5	1137.5	3	1.50
16	170	170	170	756.0	1134.0	9	0.85
17	200	200	160	680.8	1159.2	3	1.00
18	250	250	150	612.5	1137.5	6	1.25

**Table 7:** Experimental results for compressive strength.

Exp. No.	w/b	GGBS Content (%)	Fiber Content (kg/m³)	Compressive Strength (MPa)		
				28-Day Value	365-Day Value	Growth Coefficient
1	0.55	0	3	34.30	38.96	1.14
2	0.44	0	6	44.88	48.91	1.09
3	0.36	0	9	48.79	50.85	1.04
4	0.55	10	3	41.72	44.06	1.06
5	0.44	10	6	46.94	47.43	1.01
6	0.36	10	9	50.82	51.50	1.01
7	0.55	20	6	41.36	44.87	1.08
8	0.44	20	9	47.53	53.88	1.13
9	0.36	20	3	48.91	50.54	1.03
10	0.55	30	9	37.32	43.90	1.18
11	0.44	30	3	43.54	51.81	1.19
12	0.36	30	6	46.83	56.41	1.20
13	0.55	40	6	34.13	37.90	1.11
14	0.44	40	9	41.30	54.71	1.32
15	0.36	40	3	51.33	67.72	1.32
16	0.55	50	9	34.83	40.13	1.15
17	0.44	50	3	45.64	59.87	1.31
18	0.36	50	6	48.47	65.05	1.34

**Table 8:** Range analysis of compressive strength.

Factor Level	28-Day Cubic Compressive Strength (MPa)			365-Day Cubic Compressive Strength (MPa)		
	A (w/b)	B (GGBS Content)	C (Fiber Content)	A (w/b)	B (GGBS Content)	C (Fiber Content)
1	37.28	42.66	44.24	41.64	46.24	52.16
2	44.97	46.49	43.77	52.77	47.66	50.09
3	49.19	45.93	43.43	57.01	49.76	49.16
4		42.56			50.71	
5		42.26			53.44	
6		42.98			55.02	
Range value	11.92	4.24	0.81	15.37	8.78	3.00
Rank order	1	2	3	1	2	3

**Table 9:** ANOVA for 28-day compressive strength.

Variance Source	Sum of Squares	Df	Mean Square	F Value	Significance	Contribution of Factor (%)
w/b	439.001	2	219.501	44.870	0.000	79.38
GGBS content	52.789	5	10.558	2.158	0.141	5.24
Fiber content Δ	2.014	2	1.007			
Error	46.904	8	5.863			
Error Δ	48.918	10	4.892			
Total	540.709	17				

Note: The effect of fiber content is within the error.

**Table 10:** ANOVA for 365-day compressive strength.

Variance Source	Sum of Squares	Df	Mean Square	F Value	Significance	Contribution of Factor (%)
w/b	756.239	2	378.120	14.161	0.001	59.02
GGBS content	167.514	5	33.503	1.255	0.354	2.86
Fiber content Δ	28.285	2	14.143			
Error	238.724	8	29.841			
Error Δ	267.009	10	26.701			
Total	1190.763	17				

Note: The effect of fiber content is within the error.

**Table 11:** Experimental results for splitting tensile strength.

Exp. No.	w/b	GGBS Content (%)	Fiber Content (kg/m³)	Splitting Tensile Strength		Growth Coefficient
				28-Day Value	365-Day Value	
1	0.55	0	3	3.37	3.32	0.99
2	0.44	0	6	4.51	4.18	0.93
3	0.36	0	9	4.64	4.39	0.95
4	0.55	10	3	3.47	3.86	1.11
5	0.44	10	6	4.22	4.22	1.00
6	0.36	10	9	4.14	4.99	1.21
7	0.55	20	6	3.75	3.36	0.90
8	0.44	20	9	4.17	4.49	1.08
9	0.36	20	3	3.88	5.86	1.51
10	0.55	30	9	3.51	4.49	1.28
11	0.44	30	3	3.88	3.78	0.97
12	0.36	30	6	4.08	4.76	1.17
13	0.55	40	6	3.12	4.49	1.44
14	0.44	40	9	3.55	4.64	1.31
15	0.36	40	3	4.41	3.84	0.87
16	0.55	50	9	3.62	4.33	1.20
17	0.44	50	3	3.98	4.19	1.05
18	0.36	50	6	3.78	4.24	1.12

**Table 12:** Range analysis of the splitting tensile strength.

Factor Level	28-Day Splitting Tensile Strength (MPa)			365-Day Splitting Tensile Strength (MPa)		
	A (w/b)	B (GGBS Content)	C (Fiber Content)	A (w/b Ratio)	B (GGBS Content)	C (Fiber Content)
1	3.473	4.169	3.831	3.975	3.963	4.140
2	4.051	3.943	3.909	4.250	4.355	4.209
3	4.154	3.933	3.937	4.678	4.569	4.554
4		3.823			4.343	
5		3.693			4.323	
6		3.793			4.252	
Range value	0.681	0.476	0.106	0.703	0.605	0.413
Rank order	1	2	3	1	2	3

**Table 13:** ANOVA for 28-day splitting tensile strength.

Variance Source	Sum of Squares	Df	Mean Square	F Value	Significance	Contribution of Factor (%)
w/b	1.617	2	0.809	9.417	0.002	49.72
GGBS content Δ	0.405	5	0.081			
Fiber content Δ	0.036	2	0.018			
Error	0.847	8	0.106			
Error Δ	1.288	15	0.086			
Total	2.906	17				

Note: The effects of GGBS content and fiber content are within the error.

**Table 14:** ANOVA for 365-day splitting tensile strength.

Variance Source	Sum of Squares	Df	Mean Square	F Value	Significance	Contribution of Factor (%)
w/b	1.515	2	0.758	2.589	0.113	15.72
GGBS content Δ	0.582	5	0.116			
Fiber content	0.591	2	0.295	1.010	0.391	0.08
Error	3.222	8	0.403			
Error Δ	3.804	13	0.293			
Total	5.910	17				

Note: The effects of GGBS content are within the error.

**Table 15:** Experimental results for apparent chloride diffusion coefficient.

Exp. No.	w/b	GGBS Content (%)	Fiber Content (kg/m^3^)	Concentration of Sodium Chloride Solution (%)	Mean Value of *D_app_* (×10^−12^ m^2^/s)
1	0.55	0	3	3.5	9.03
2	0.44	0	6	5	8.90
3	0.36	0	9	7	7.07
4	0.55	10	3	5	12.87
5	0.44	10	6	7	7.17
6	0.36	10	9	3.5	3.67
7	0.55	20	6	7	11.66
8	0.44	20	9	3.5	5.59
9	0.36	20	3	5	3.10
10	0.55	30	9	5	10.96
11	0.44	30	3	7	5.28
12	0.36	30	6	3.5	3.51
13	0.55	40	6	3.5	8.64
14	0.44	40	9	5	4.42
15	0.36	40	3	7	3.30
16	0.55	50	9	7	10.53
17	0.44	50	3	3.5	3.73
18	0.36	50	6	5	1.51

**Table 16:** Range analysis of the chloride diffusion coefficient.

Factor Level	$\mathrm{Chloride}\;\mathrm{Diffusion}\;\mathrm{Coefficient}\;\mathrm{after}\;24\;\mathrm{Drying}–\mathrm{Wetting}\;\mathrm{Cycles}\;(\times 10^{-12}\;m^{2}/s$)			
	A (w/b)	B (GGBS Content)	C (Fiber Content)	D (Concentration of Sodium Chloride Solution)
1	10.614	8.332	6.219	5.695
2	5.849	7.905	6.898	6.961
3	3.693	6.783	7.040	7.501
4		6.584		
5		5.453		
6		5.255		
Range value	6.921	3.077	0.821	1.806
Rank order	1	2	4	3

**Table 17:** ANOVA results for $D_{app}$.

Variance Source	Sum of Squares	Df	Mean Square	F Value	Significance	Contribution of Factor (%)
w/b	150.521	2	75.261	43.830	0.000	74.32
GGBS content	23.328	5	4.666	2.717	0.101	7.45
Fiber content Δ	2.312	2	1.156			
Solution concentration	10.314	2	5.157	3.003	0.106	3.48
Error	11.425	6	1.904			
Error Δ	13.737	8	1.717			
Total	197.900	17				

Note: The effect of fiber content is within the error.

**Table 18:** SNR values of each group.

Exp. No.	SNR Value				
	28-Day Compressive Strength	365-Day Compressive Strength	28-Day Splitting Tensile Strength	365-Day Splitting Tensile Strength	$D_{app}$
1	30.68	31.81	10.54	10.43	−19.12
2	33.02	33.79	13.07	12.43	−18.99
3	33.76	34.13	13.32	12.84	−16.98
4	32.40	32.88	10.81	11.73	−22.19
5	33.43	33.52	12.51	12.51	−17.11
6	34.11	34.24	12.34	13.96	−11.30
7	32.33	33.04	11.48	10.53	−21.34
8	33.54	34.63	12.40	13.04	−14.95
9	33.78	34.07	11.78	15.35	−9.82
10	31.44	32.85	10.91	13.04	−20.80
11	32.77	34.29	11.78	11.55	−14.46
12	33.39	35.03	12.21	13.55	−10.91
13	30.64	31.57	9.88	13.04	−18.73
14	32.30	34.76	11.00	13.33	−12.92
15	34.21	36.61	12.89	11.68	−10.37
16	30.84	32.07	11.17	12.73	−20.44
17	33.16	35.54	12.00	12.44	−11.43
18	33.68	36.26	11.55	12.55	−3.59

## Data Availability

The original contributions presented in the study are included in the article, further inquiries can be directed to the corresponding author.
